# Optical Coherence Tomography for Recurrent Drug Eluting Stent-Related In-Stent Restenosis: Stepwise Intracoronary Imaging of Progressive Tissue and Calcium Modification

**DOI:** 10.1016/j.jscai.2025.104115

**Published:** 2025-12-30

**Authors:** Evan Harmon, Laura Young, Khaled Ziada

**Affiliations:** Department of Cardiovascular Medicine, Heart, Vascular and Thoracic Institute, Cleveland Clinic, Cleveland, Ohio

**Keywords:** drug-eluting stents, in-stent restenosis, intravascular lithotripsy, optical coherence tomography

## Case report

An 86-year-old woman presented for percutaneous coronary intervention (PCI) to the right coronary artery (RCA) due to recurrent in-stent restenosis (ISR). Ten months earlier, she presented with NSTEMI and was found to have a severely calcified culprit ostial RCA stenosis with otherwise moderate diffuse disease of her left coronary system. She underwent intravascular ultrasound–guided PCI to the ostial/proximal RCA, which was noted to be severely calcified, with intravascular lithotripsy (IVL) and stenting with a 3.5 mm × 38 mm everolimus-eluting stent.

Three months later, she presented with another NSTEMI and was found to have severe ISR in the proximal RCA stent. That was treated with a 3.75 mm cutting balloon with a good angiographic result. Unfortunately, 3 months later, she developed anginal recurrence and underwent PET stress testing, demonstrating moderate RCA territory ischemia. Subsequent angiography revealed recurrent critical proximal RCA ISR; hence, she was transferred to our institution for potential advanced therapies for recurrent ISR.

**Figure 1:**
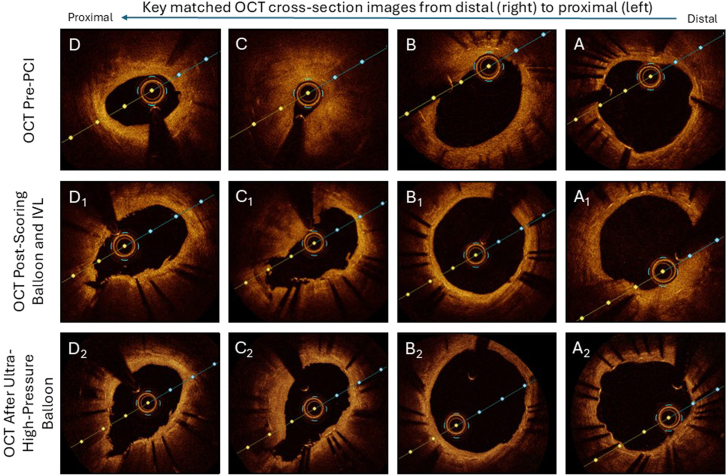
**Key optical coherence tomography (OCT) findings.** Panels (**A**), (**B**), (**C**), and (**D**) correspond to the distal stent region, the midstent region, the region of minimal stent area (MSA), and the proximal stent region, respectively. Prior to percutaneous coronary intervention (PCI), we identified severe heterogeneous neointimal hyperplasia (NIH) with MSA 0.87 mm^2^ (C). Following scoring balloon angioplasty and intravascular lithotripsy (IVL), MSA increased to 4.86 mm^2^ (C_1_). Treatment of the ostial/proximal segment with ultrahigh-pressure balloon angioplasty further increased MSA to 6.02 mm^2^ (C_2_).

As we embarked on her procedure, a primary objective was to understand the mechanism of recurrent stent failure. After predilation with a small balloon, we performed optical coherence tomography (OCT) which demonstrated extensive heterogeneous neointimal hyperplasia (NIH) with minimal stent area (MSA) of 0.87 mm^2^ (Figure 1A-D and Supplemental Video 1), relative stent expansion 41.7%, a single layer of stent struts, and heavy calcification of the native vessel outside the stent (Supplemental Video 1). Thus, stent failure was attributed to both excess NIH as well as underexpansion caused by calcification. Interestingly, there was also evidence of possible mild longitudinal stent deformation (Supplemental Video 1, Frame 169), which may have been contributing as well.

**Figure 2:**
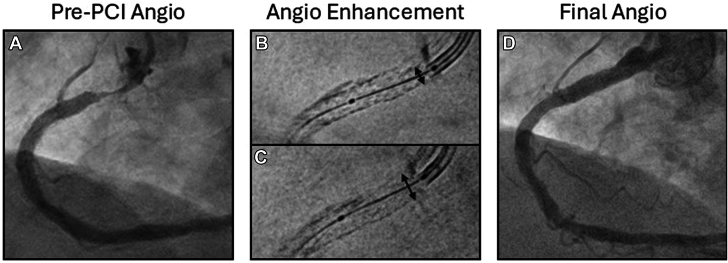
**Angiographic findings and visualization of stent underexpansion.** Pre-PCI angiography demonstrated critical ostial in-stent restenosis (**A**). Stent-enhancing technology demonstrated both native vessel calcification as well as a narrow stent diameter (**B**). Following ultrahigh-pressure balloon inflation at 40 atm, stent expansion improved dramatically (**C**). Final angiography after repeat stenting demonstrated excellent stent expansion (**D**). PCI, percutaneous coronary intervention.

We used a 4.0 mm scoring balloon to modify the NIH, followed by IVL (4.0 mm balloon, 120 pulses delivered). Repeat OCT demonstrated modification of the neointima with MSA of 4.86 mm^2^ and relative stent expansion 43.8% (Figure 1A_1_-D_1_ and Supplemental Video 2). However, the proximal edge of the stent remained underexpanded due to vessel calcification (Figure 2A), confirmed by cineangiographic stent enhancement technology (Figure 2B). The proximal edge was then treated using a 3.5 mm × 15 mm ultrahigh-pressure balloon at 40 atm, resulting in improved expansion (Figure 2C).

Subsequent imaging demonstrated an MSA of 6.02 mm^2^ with relative stent expansion of 68.8% (Figure 1A_2_-D_2_ and Supplemental Video 3). We stented the lesion with a 4.0 mm × 16 mm everolimus-eluting stent, which was subsequently postdilated with a 4.0 mm noncompliant balloon. Final OCT demonstrated adequate stent expansion and apposition with MSA >9 mm^2^ and relative stent expansion of 98.1% (Supplemental Video 4), and final angiography (Figure 2D) demonstrated an excellent result with no evidence of dissection or perforation. She was discharged home the following day, and remains asymptomatic with no recurrence of her previously life-limiting angina as of 4 months postprocedure.

## Discussion

Intracoronary imaging has consistently been shown to improve outcomes following PCI, leading to strong recommendations for its routine utilization in both acute and chronic coronary syndromes.^1^^,^^2^ Recurrent ISR is a commonly encountered problem for which intracoronary imaging is especially useful. Even in the modern era of PCI, the rate of ischemia-driven target lesion revascularization remains 2% per year, and ISR accounts for 10% of PCI in the United States.^3^ The appropriate use and interpretation of the 2 commonly used intracoronary imaging modalities, intravascular ultrasound and OCT, is critical in the evaluation and treatment of ISR.

In this case, OCT imaging demonstrated more than one mechanism of stent failure in a single ISR lesion. Though poor visualization of aorto-ostial lesions is an important potential limitation of OCT-guided PCI, we believe that OCT overall provides several key advantages in cases of recurrent ISR. First, the greater axial resolution of OCT allows for: (1) delineation of the lumen-neointimal interface, (2) identification of both NIH and neointimal calcification, and (3) clear visualization of stent layers.^3^^,^^4^ Additionally, the near-infrared light source of OCT is able to penetrate beyond calcium, which allows for quantification of both neointimal and native vessel calcification thickness, arc of distribution, and area.^4^ The degree of NIH, the presence or absence of neointimal and/or native vessel calcification, the extent, thickness, and morphology (ie, sheets vs nodules) of that calcification, and the number of stent layers all have important implications in the selection of treatment modalities for ISR. Prognostically, factors including neointimal and peri-stent calcification as well as multiple stent layers have been shown to increase the risk of new stent underexpansion and higher rates of myocardial infarction and target vessel revascularization.^5^

In our case, the use of OCT dynamically changed our treatment strategy. Our initial OCT run demonstrated dense, heterogeneous NIH with components of both neointimal and peri-stent calcification. The target lesion in this case could be classified as a Waksman type IIA lesion (predominantly NIH).^6^ As such, we first chose to treat using a scoring balloon, which minimizes the “watermelon seeding” phenomenon of balloon angioplasty within focal ISR lesions, resulting in less geographic miss and improved acute luminal gain.^7^ Additionally, scoring balloon angioplasty improves angiographic outcomes for drug-eluting stent (DES)–related ISR prior to treatment with drug-coated balloon (DCB) therapy at 6 to 8 months.^8^

Following the use of scoring balloon angioplasty, we favored the use of IVL to address the neointimal and peri-stent calcification observed on OCT. Though the safety and efficacy of IVL in de novo calcific lesions have been well-described, its use in ISR lesions remains off-label. However, a recent meta-analysis of 354 patients with stent underexpansion due to calcified plaque demonstrated a procedural success rate (defined as residual angiographic stenosis <30% or <20% depending on the included study) of 88.7% with very low complication rates (<2.0%).^9^ Interestingly, the lone factor associated with a positive influence on procedural success was predilation with ultrahigh-pressure balloon prior to IVL. In our case, we only first noted significant proximal stent underexpansion on serial OCT imaging following scoring balloon angioplasty and IVL, which we confirmed by stent enhancement technology, leading to our use of an ultrahigh-pressure balloon thereafter. Importantly, stent underexpansion was found to be a major driver of adverse clinical outcomes in the RESTO registry.^10^

A last decision point in this case was the use of DES vs DCB as a final treatment strategy. Overall, most randomized trials and meta-analyses have demonstrated a clear benefit to repeat DES implantation over DCB for treatment of DES-related ISR.^7^ Though limited data have previously suggested improved angiographic and clinical outcomes when switching from one DES to another type of drug in cases of DES-ISR,^11^ this remains controversial. Given our patient had one stent layer, and that the multiple underlying etiologies of ISR were adequately addressed, we chose to perform repeat stenting with an everolimus-eluting stent.

## Conclusion

Treatment of recurrent DES-ISR remains a significant challenge. OCT imaging can provide critical insight into the mechanism of ISR in these cases, dramatically impacting treatment strategy.

## Declaration of competing interest

The authors declared no potential conflicts of interest with respect to the research, authorship, and/or publication of this article.

## References

[1] Vrints C., Andreotti F., Koskinas K.C. (2024). 2024 ESC Guidelines for the management of chronic coronary syndromes. Eur Heart J.

[2] Rao S.V., O’Donoghue M.L., Ruel M. (2025). 2025 ACC/AHA/ACEP/NAEMSP/SCAI guideline for the management of patients with acute coronary syndromes: a report of the American College of Cardiology/American Heart Association Joint Committee on Clinical Practice Guidelines. Circulation.

[3] Giustino G., Colombo A., Camaj A. (2022). Coronary in-stent restenosis: *JACC* State-of-the-Art Review. J Am Coll Cardiol.

[4] Maehara A., Matsumura M., Ali Z.A., Mintz G.S., Stone G.W. (2017). IVUS-guided versus OCT-guided coronary stent implantation: a critical appraisal. JACC Cardiovasc Imaging.

[5] Yin D., Mintz G.S., Song L. (2020). In-stent restenosis characteristics and repeat stenting underexpansion: insights from optical coherence tomography. EuroIntervention.

[6] Shlofmitz E., Iantorno M., Waksman R. (2019). Restenosis of drug-eluting stents: a new classification system based on disease mechanism to guide treatment and state-of-the-art review. Circ Cardiovasc Interv.

[7] Alfonso F., Coughlan J.J., Giacoppo D., Kastrati A., Byrne R.A. (2022). Management of in-stent restenosis. EuroIntervention.

[8] Kufner S., Joner M., Schneider S. (2017). Neointimal modification with scoring balloon and efficacy of drug-coated balloon therapy in patients with restenosis in drug-eluting coronary stents: a randomized controlled trial. JACC Cardiovasc Interv.

[9] Caminiti R., Vetta G., Parlavecchio A. (2023). A systematic review and meta-analysis including 354 patients from 13 studies of intravascular lithotripsy for the treatment of underexpanded coronary stents. Am J Cardiol.

[10] Souteyrand G., Mouyen T., Honton B. (2024). Stent underexpansion is an underestimated cause of intrastent restenosis: insights from RESTO registry. J Am Heart Assoc.

[11] Alfonso F., Pérez-Vizcayno M.J., Dutary J. (2012). Implantation of a drug-eluting stent with a different drug (switch strategy) in patients with drug-eluting stent restenosis. Results from a prospective multicenter study (RIBS III [restenosis intra-stent: balloon angioplasty versus drug-eluting stent]). JACC Cardiovasc Interv.

